# Update on the efficacy and safety of intravenous tranexamic acid in hip fracture surgery: a systematic review and meta-analysis

**DOI:** 10.1007/s00590-022-03387-9

**Published:** 2022-09-26

**Authors:** Shahid Miangul, Timothy Oluwaremi, Joe El Haddad, Maamoun Adra, Nathan Pinnawala, Hayato Nakanishi, Reem H. Matar, Christian A. Than, Thomas M. Stewart

**Affiliations:** 1 St George’s University of London, London, SW17 0RE UK; 2 University of Nicosia Medical School, University of Nicosia, 2417 Nicosia, Cyprus; 3 Department of Gastroenterology and Hepatology, Mayo Clinic, Rochester, MN USA; 4 School of Biomedical Sciences, The University of Queensland, St Lucia, Brisbane, 4072 Australia; 5 Department of Anesthesiology and Perioperative Medicine, Mayo Clinic, Rochester, MN USA

**Keywords:** Hip fracture surgery, Tranexamic acid, TXA, Blood loss, Meta-analysis

## Abstract

**Aim:**

The aim of this meta-analysis was to assess the safety and efficacy of tranexamic acid (TXA) in the management of hip fracture surgeries in comparison with placebo.

**Methods:**

A systematic search was conducted from August 6, 2021. Eligible studies included randomized clinical trials and prospective studies comparing the use of intravenous TXA in patients treated for hip fractures, in comparison with placebo. Review Manager was used for the meta-analysis.

**Results:**

Eighteen prospective studies including 14 RCTs met the eligibility criteria. The results favored the TXA group in the quantity of total blood loss (MD =  − 196.91 mL, 95% CI − 247.59, − 146.23, *I*^2^ = 92%), intraoperative blood loss (MD = − 26.86 mL, 95% CI − 36.96, − 16.78, *I*^2^ = 62%), and rate of blood transfusion (OR 0.35, 95% CI 0.28, 0.42, *I*^2^ = 0%). TXA also exhibited higher hemoglobin level at day 1 (MD = 6.77 g/L, 95% CI 4.30, 9.24, *I*^2^ = 83%) and day 3 (MD = 7.02 g/L, 95% CI 3.30, 10.74, *I*^2^ = 82%) postoperatively. There was no significant difference found in the incidence of thromboembolic events from occurring between the two groups, such as deep vein thrombosis (OR 1.22, 95% CI 0.73, 2.02, *I*^2^ = 0%) and pulmonary embolism (OR 0.82, 95% CI 0.33, 2.05, *I*^2^ = 0%).

**Conclusion:**

Administration of intravenous TXA appears to reduce blood loss, rate of blood transfusions and pose no increased risk of thromboembolic events. Therefore, TXA should be considered by physicians when managing hip fracture patients.

**Graphical abstract:**

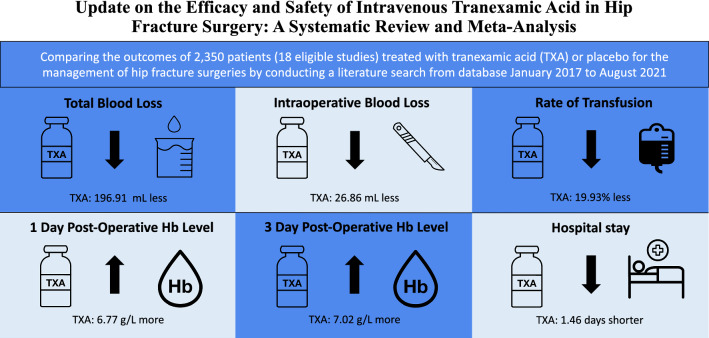

**Supplementary Information:**

The online version contains supplementary material available at 10.1007/s00590-022-03387-9.

## Introduction

Hip fractures have a reported prevalence rate of 10% in men and 20% in women [1] and can expect to reach an incidence of 6.3 million by the year 2050 [2]. As life expectancy rises around the world, hip fractures will continue to account for a significant public health burden. These injuries can frequently lead to disability and in more severe cases, mortality within the elderly population with an estimated 20% mortality rate in people over the age of 65 year within the first year of postoperative discharge [3].

The primary management for hip fractures is to perform surgery within 24 h, with earlier management proving to result in better outcomes and reduced perioperative complications and mortality [4]. One complication that can arise during the surgical management of hip fractures is poor hemostatic control. Tranexamic acid (TXA) is a pharmacological agent that is commonly used to control the amount of blood loss during surgical procedures. Being a synthetic derivative of the amino acid lysine, TXA acts as an anti-fibrinolytic by reversibly inhibiting binding sites of lysine on plasminogen, thus preventing the degradation of formed clots [5]. Clinically, TXA is a cost-effective drug that can be administered both intravenously and topically [6]. An important factor in considering TXA is related to the point at which the rate of fibrinolysis for total knee and hip arthroplasty has shown to peak, which is estimated around 6 h after surgery and can be maintained for up to 18 h [7]. By countering fibrinolysis during this peak interval, the use of TXA perioperatively can help to inhibit the activation of plasmin and effectively reduce blood loss. TXA has already proven to reduce blood loss in many orthopedic surgeries, which include knee arthroplasty [8, 9], humeral fractures [10] as well as pelvic and acetabular fractures [11, 12]. Importantly, previous meta-analyses have explored the safety of TXA in total hip arthroplasties and have shown to reduce allogeneic transfusion rates [13], decrease total and intraoperative blood loss [14] as well as lower postoperative hemoglobin decline [15], when compared to the placebo. One area of concern with TXA is the susceptibility for abnormal clotting, in which Myers et al. [16] cautioned TXA as a potential independent risk factor for post-traumatic venous thromboembolism. However, in continuation with findings from randomized clinical trials, meta-analyses conducted have found no increased risk in thromboembolic events taking place between the TXA intervention and placebo groups for hip arthroplasties [13–15].

This meta-analysis aims to examine and extend the findings found in the literature by incorporating recent evidence-based studies to ultimately facilitate guidelines for the clinical use of TXA in hip fracture surgeries. By examining multiple randomized clinical trials (RCTs) and prospective studies, this meta-analysis can be useful in helping physicians to understand and consider the use of TXA as a preventative modality to reduce blood loss and the onset of postoperative complications, when managing patients undergoing hip fracture surgeries.

## Methods

### Data sources and search strategies

A comprehensive search of several databases from inception to August 6, 2021 was conducted in compliance with the Preferred Reporting Items for Systematic Reviews and Meta-analyses (PRISMA) guidelines [17]. The databases included Ovid MEDLINE(R) and Epub Ahead of Print, PubMed, In-Process and Other Non-Indexed Citations and Daily, Ovid Embase, Ovid Cochrane Central Register of Controlled Trials, Ovid Cochrane Database of Systematic Reviews and Scopus. The search strategy was designed and conducted by an experienced librarian with input from the study’s principal investigator. Controlled vocabulary supplemented with keywords was used to search for studies describing tranexamic acid in hip fracture. The actual strategy listing all search terms used and how they are combined is available in Supplementary Item 1.

### Eligibility criteria and quality assessment

Eligible studies were RCTs or prospective cohort studies that must meet all of the following inclusion criteria: (1) Comparative studies of adult participants older than or equal to 18 years who underwent hip surgery with either intravenous tranexamic acid or placebo for the treatment of hip fracture; (2) Outcomes of total blood loss; and/or (3) Outcomes of transfusion rate. (4) Selected studies with publication date from 2017 onwards. Reports, case series, conference abstracts and/or abstracts, and articles that could not be translated to English were excluded from the study. Articles that were of any language other than English were translated using google translate. The quality of each study was independently evaluated by two authors (SM and TO) using the Cochrane Handbook to formulate the ‘risk of bias’ table [18]. Results of the quality assessment of all included studies are shown in Supplementary Item 2.

### Statistical analysis

The pooled means and proportions of our data were analyzed using an inverse variance method for continuous data and Mantel–Haenszel method for dichotomous data, which assigns the weight of each study based on its variance. A direct comparison between the two techniques was conducted by assessing studies that reported outcomes of both treatments (two-arm analysis). The heterogeneity of effect size estimates across the studies was quantified using the Q statistic and *I*^2^. A value of *I*^2^ of 0–25% indicates insignificant statistical heterogeneity, 26–50% low heterogeneity, 51–100% high heterogeneity [19]. The Random effects model was used when the value of *I*^2^ was > 50% and the fixed effects model was used for *I*^2^ < 50%. If mean and standard deviation (SD) were not available, the median was converted to mean using the formulas from the Cochrane Handbook for Systematic Reviews of Interventions [20]. Additionally, if mean and SD were only depicted in figures, mean and SD were digitized from figures using WebPlotDigitizer version 4.4 (https://automeris.io/WebPlotDigitizer/). Data analysis was performed using RevMan software version 5.4 (Review Manager (RevMan) [Computer program]. The Cochrane Collaboration, 2020, Copenhagen, Denmark).

## Results

### Study selection and patient characteristics

The initial literature search of electronic databases resulted in 438 total studies. After the removal of duplicates, the articles were screened for inclusion and exclusion criteria, 54 studies were evaluated, and full texts were assessed for eligibility. Eighteen studies (*n* = 2350, 63.4% female) met the eligibility criteria and were included in this meta-analysis (Supplementary Item 1). The majority of the eligible studies were randomized clinical trials (*n* = 14). Four studies were non-RCT prospective cohort studies. The date of publication of the included studies ranged between 2017 and 2021. Among the overall population, 1178 patients were administered the placebo, and 1172 patients were administered TXA. Supplementary Item 3 illustrates the details of the study selection process. The baseline characteristics of the included studies are detailed in Table 1. The age of combined participants at the time of surgery ranged from 65.7 to 84.3 years.

**Table 1:** Baseline characteristic of included studies

Study	Publication year	Study type	Total number of participants (C/TXA)	Gender (M/F)	Control mean age (years)	TXA mean age (years)	TXA dose	Placebo used
Ahmed et al.	2020	RCT	120 (60/60)	59/61	65.87 ± 8.60	65.60 ± 8.17	15 mg/kg preoperatively	Saline (same dose)
AlSumadi et al.	2021	Prospective Cohort Study	613 (306/307)	171/441	82.00 ± 8.80	82.00 ± 8.50	1 g on induction of anesthesia (1 dose)	NR
Chen et al.	2019	RCT	176 (88/88)	76/100	77.40 ± 6.80	76.80 ± 7.00	15 mg/kg in 100 mL of saline 15 min before, during surgery and 3 h after surgery (3 doses)	Saline (100 mL)
Hao et al.	2020	RCT	65 (32/33)	20/45	72.25 ± 7.65	75.15 ± 9.36	1 g 30 min before operation	Equal volume of physiological saline
Huang et al.	2021	RCT	156 (78/78)	83/73	76.90 ± 5.40	77.70 ± 5.60	20 mg/kg 30 min before surgery (1 dose)	NR
Lei et al.	2017	RCT	77 (40/37)	12/65	79.20 ± 6.50	77.80 ± 9.80	1 g in 200 mL of saline before surgery (1 dose)	Saline (200 mL)
Luo et al.	2019	RCT	90 (46/44)	43/47	76.10 ± 9.30	75.10 ± 8.00	15 mg/kg 15 min before and 3 h after surgery (2 doses)	Saline (100 mL)
Narkbunnam et al.	2021	RCT	60 (30/30)	16/44	73.10 ± 5.25	78.80 ± 6.50	750 mg (250 mg/5 mL) before skin incision	Normal saline (20 mL)
Nikolaou et al.	2021	RCT	165 (88/77)	41/124	83.40 ± 12.00	82.90 ± 8.80	15 mg/kg in 100 mL saline 5 min before surgery (1 dose)	Saline (100 mL)
Qingyan et al.	2020	RCT	30 (15/15)	15/15	73.10 ± 5.25	71.20 ± 6.00	1 g before operation	Saline (100 mL)
Qiu et al.	2020	Prospective Cohort Study	80 (40/40)	43/37	66.90 ± 5.80	68.20 ± 7.30	15 mg/kg 20 min before surgery (1 dose)	Saline (15 mg/kg)
Schiavone et al.	2018	RCT	90 (43/47)	27/63	84.30 ± 8.30	84.30 ± 8.30	15 mg/kg at time of surgical incision (1 dose)	Saline (15 mg/kg)
Tian et al.	2018	RCT	100 (50/50)	33/67	79.30 ± 6.60	77.74 ± 6.50	10 mg/kg 10 min before surgery and 5 h after surgery (2 doses)	NR
Watts et al.	2017	RCT	138 (69/69)	43/95	82.20 ± 10.00	81.00 ± 10.00	15 mg/kg at time of surgical incision and at wound closure, administered over 10 min (2 doses)	Saline (100 mL)
Zhang et al.	2020	RCT	122 (61/61)	62/60	76.10 ± 16.60	79.10 ± 11.90	1 g in 100 mL of saline 10 min before surgery and 3 h after surgery (2 doses)	Saline (100 mL)
Zhaopeng et al.	2020	Prospective Cohort Study	60(30/30)	24/36	82.57 ± 7.87	79.70 ± 7.40	0.5 g within 30 min before incision	100 mL 5% glucose solution
Zhi-Chao et al.	2018	Prospective Cohort Study	108(52/56)	41/55	72.33 ± 7.03	72.13 ± 6.88	1 g in 250 mL of physiological saline, preoperatively	Normal saline (250 mL)
Zhou et al.	2019	RCT	100 (50/50)	37/63	77.80 ± 6.40	75.10 ± 8.27	1 g in 100 mL 15 min before surgery (1 dose)	NR

*C* Control group, *F* Female, *M* Male, *NR* Not Reported, *RCT* Randomized clinical trial, *TXA* Tranexamic acid group

### Risk of bias

In the Supplementary Item 2, the results of the quality assessment for the studies included are shown. The RCT and prospective studies that were selected were judged to be of good quality. The patients appeared to represent the whole experience of the investigator, and the exposure and outcome were adequately ascertained. The lengths of follow-up were adequate.

### Clinical and general characteristics of the studies included

Eighteen studies met eligibility criteria with a total of 2350 participants being administered either the placebo or tranexamic acid (TXA), of which 60.70% and 62.03% of patients were female in the placebo and TXA group, respectively. The mean body mass index (BMI) was calculated as 23.25 kg/m^2^ for the placebo group and 23.51 kg/m^2^ for the TXA group, with no significant difference between the two (MD = 0.27 kg/m^2^, 95% CI − 0.02, 0.56, *I*^2^ = 0%). In the placebo group, the preoperative comorbidities that were reported in patients consisted of 123 patients with hypertension, 71 with diabetes, 84 with cardiac disease and 22 with pulmonary disease. In the TXA group, there were 123 patients with hypertension, 79 with diabetes, 84 with cardiac disease and 20 with pulmonary disease (Table 2). The most common type of hip fracture patients experienced was intertrochanteric fractures with 1179 events (598 and 581 in the placebo and TXA groups, respectively) among the entire cohort. The second most common was femoral neck fractures with a total of 262 (128 and 134 in the placebo and TXA groups, respectively), and the third most common was trochanteric fractures with a total of 176 (88 and 88 in the placebo and TXA groups, respectively) (Table 2). The length of operation time was reported in ten studies. The TXA group reported a mean operative time (mins) of 79.99 ± 30.96 min, while the placebo reported 80.30 ± 32.07 days. There was no difference in the pooled operative length between the placebo and TXA groups (MD = − 0.87 min, 95% CI − 2.06, 0.32, *I*^2^ = 0%). In this meta-analysis, seven of the eighteen studies used a fixed dose of 1 g of TXA, while the other studies used a weighted dose/s ranging from 10 mg/kg, 15 mg/kg, and 20 mg/kg in saline solution, as summarized in Table 1. The solution administered to the placebo group was primarily saline or otherwise not reported in the studies included for this meta-analysis.

**Table 2:** Reported comorbidities and hip fracture types

	Placebo group	TXA group
*Comorbidities*		
Hypertension (*n*)	123	123
Diabetes (*n*)	71	79
Cardiac disease (*n*)	84	84
Neurological disease (*n*)	11	10
Pulmonary disease (*n*)	22	20
Urological disease (*n*)	17	11
*Type of fracture*		
Intertrochanteric	598	581
Trochanteric	88	88
Pre-trochanteric	43	47
Femoral neck	128	134

### Total blood loss

**Fig. 1:**
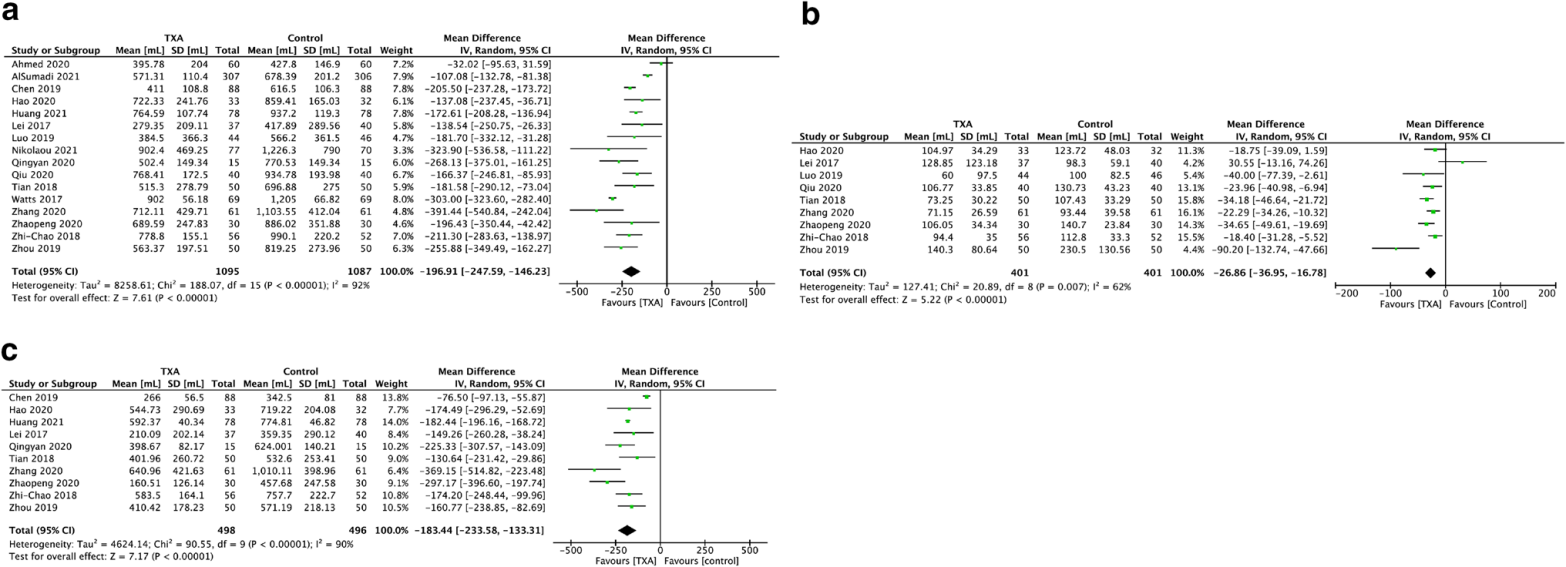
Pooled estimate of blood loss. **a** Total blood loss, **b** Intraoperative blood loss, **c** Hidden blood loss

Sixteen studies including 2182 patients reported the total blood after surgery was completed for hip fractures. One thousand ninety-five patients were treated with TXA and 1087 with placebo (Fig. 1a). Due to heterogeneity, a random effects model was applied. The mean total blood loss for the TXA group was 618.91 mL, compared to the placebo group which reported 807.30 mL (Table 3). There was reduced blood loss in the TXA group when compared to the placebo group, with a mean difference of 196.91 mL favoring TXA group (95% CI − 247.59, − 146.23, *I*^2^ = 92%).

**Table 3:** Intraoperative and postoperative outcomes

Outcomes	Placebo group	TXA group
Total blood loss (mL)	807.30 ± 389.75	618.91 ± 282.72
Hidden blood loss (mL)	619.47 ± 311.93	438.86 ± 265.61
Intraoperative blood loss (mL)	136.09 ± 86.19	103.60 ± 75.75
Transfusion rate (*n*)	456 (*n* = 1118)	232 (*n* = 1112)
Postoperative Hb Level day 1 (g/L)	95.55 ± 28.17	104.42 ± 13.72
Postoperative Hb Level day 3 (g/L)	84.30 ± 17.79	91.46 ± 14.74
Incidence of postoperative DVT (*n*)	22 (*n* = 753)	27 (*n* = 753)
Incidence of postoperative PE (*n*)	28 (*n* = 830)	34 (*n* = 831)
Length of hospital stay (days)	11.19 ± 5.80	10.13 ± 4.11
Operation time (minutes)	80.30 ± 32.07	79.99 ± 30.96

*DVT* Deep vein thrombosis, *Hb* Hemoglobin, *PE* Pulmonary embolism

### Intraoperative blood loss

From nine studies, 802 patients reported intraoperative blood loss. Four hundred and one patients were treated with TXA and 401 with placebo (Fig. 1b). Due to heterogeneity, a random effects model was applied. The mean total blood loss for the TXA group was 103.60 mL, compared to the placebo group which reported 136.09 mL (Table 3). With random effects model, there was a reduced intraoperative blood loss between the two groups, favoring TXA group (MD = − 26.86 mL, 95% CI − 36.96, − 16.78, *I*^2^ = 62%).

### Hidden blood loss

From ten studies, 994 patients reported hidden blood loss. Four hundred and ninety-eight patients were treated with TXA and 496 with placebo (Fig. 1c). Due to heterogeneity, a random effects model was applied. The mean total blood loss for the TXA group was 438.86 mL, compared to the placebo group which reported 619.48 mL (Table 3). With random effects model, there was a reduced hidden blood loss between the two groups, favoring TXA group (MD = − 183.44 mL, 95% CI − 233.58, − 133.31, *I*^2^ = 90%).

### Transfusion rate

**Fig. 2:**
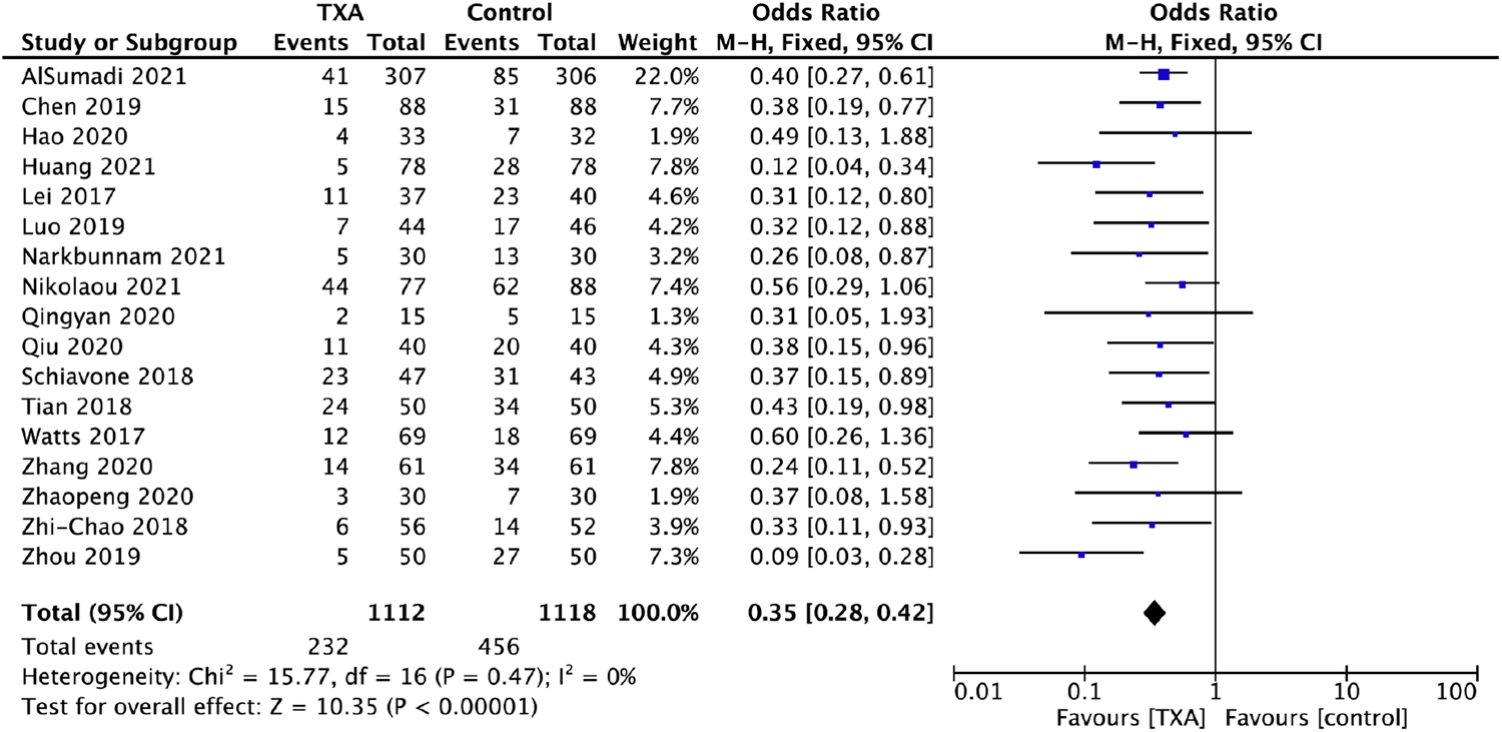
Pooled estimate of transfusion rates

Seventeen studies involving 2230 patients reported the rate of blood transfusion. One thousand one hundred twelve patients were treated with TXA and 1118 with placebo (Fig. 2). There was no difference in heterogeneity, and a fixed effects model was applied. A decreased transfusion rate was detected in the TXA group, whereby 232 of 1112 (20.86%) patients were transfused, when compared to the placebo group, whereby 456 of 1118 (40.79%) patients received blood transfusion (OR 0.35, 95% CI 0.28, 0.42, *I*^2^ = 0%).

### Hospital stay

**Fig. 3:**
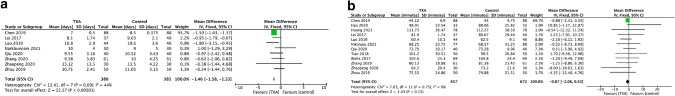
Pooled estimate of hospital stay and operation time. **a** Hospital stay, **b** Operative time

Eight studies involving 765 patients reported on the length of hospital stay after surgery. Three hundred and eighty patients were treated with TXA and 385 with placebo (Fig. 3a). A fixed effects model was applied. The TXA group had reduced length of hospital stay with a mean of 11.19 days, when compared to the placebo group which reported a mean of 10.13 days (MD = − 1.46 days, 95% CI − 1.58, − 1.33, *I*^2^ = 44%).

### Postoperative Hb levels (Day 1 and 3)

**Fig. 4:**
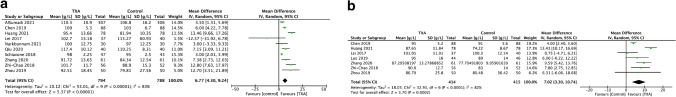
Pooled estimate of postoperative hemoglobin levels. **a** Day 1, **b** Day 3

Ten studies involving 1582 patients reported postoperative hemoglobin concentration at day 1. Seven hundred and ninety-four patients were treated with TXA and 788 with placebo (Fig. 4a). At day 1, the mean Hb level of the TXA group was 104.42 g/L, whereas the placebo group Hb level was 95.55 g/L (Table 3). A random effects model was applied due to heterogeneity. With random effects model, pooled postoperative Hb level at day 1 was favorable in the TXA group (Fig. 4a), when compared to the placebo group (MD = 6.77 g/L, 95% CI 4.30, 9.24, *I*^2^ = 83%).

Seven studies involving 829 patients reported postoperative Hb concentration at day 3. Four hundred and fourteen patients were treated with TXA and 415 with placebo (Fig. 4b). At day 3, the mean Hb level of the TXA group was 91.46 g/L, whereas the placebo group Hb level was 84.30 g/L (Table 3). A random effects model was applied due to heterogeneity. With random effects model, pooled postoperative Hb level at day 3 was favorable in the TXA group (Fig. 4b), when compared to the placebo group (MD = 7.02 g/L, 95% CI 3.30, 10.74, *I*^2^ = 82%).

### Postoperative complications

#### Deep vein thrombosis (DVT)

**Fig. 5:**
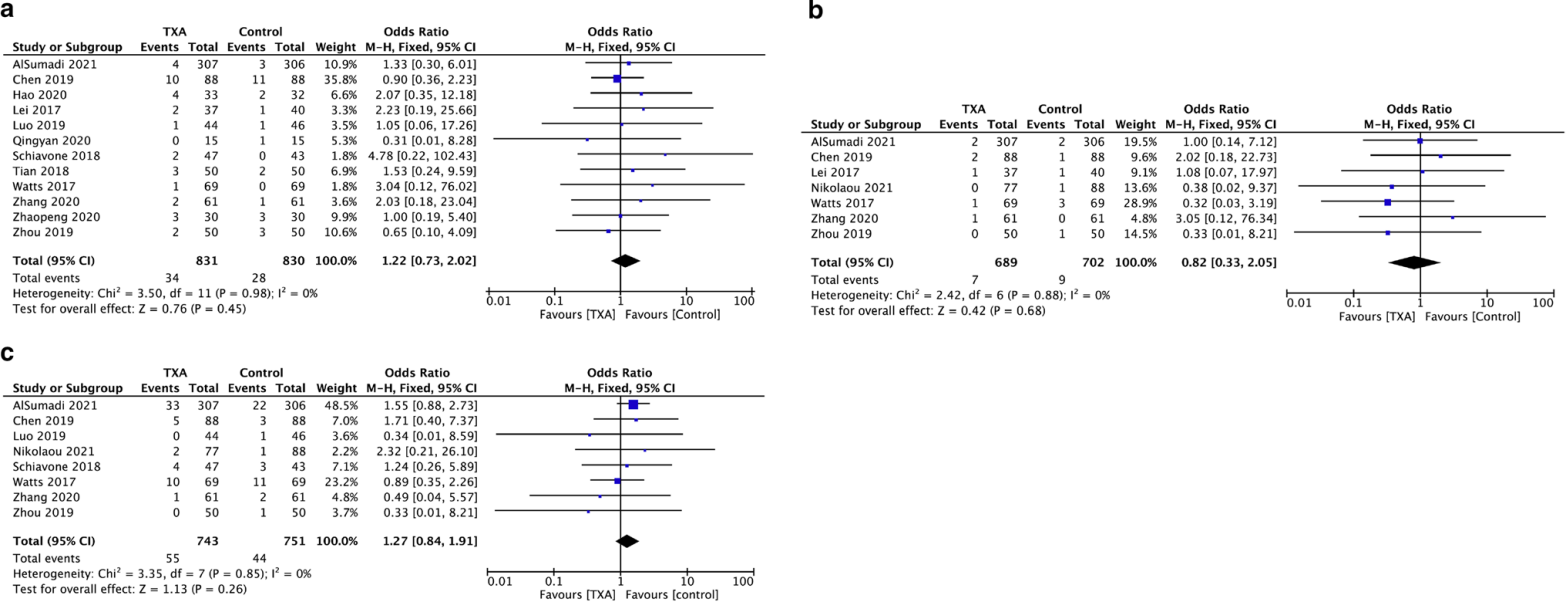
Pooled estimate of postoperative incidences. **a** Deep vein thrombosis, **b** Pulmonary embolism, **c** Mortality

Twelve studies involving 1661 patients reported on the rates of deep vein thrombosis (DVT). Eight hundred and thirty-one patients were treated with TXA and 830 with placebo (Fig. 5a). Of the 831 patients in the TXA group, 34 were reported to have incidence of DVT, whereas 28 of the 830 patients in the placebo group had incidence of DVT. A fixed model was applied. There was no difference between TXA and placebo groups in occurrence of DVT (OR 1.22, 95% CI 0.73, 2.02, *I*^2^ = 0%).

#### Pulmonary embolism (PE)

Seven studies involving 1391 patients reported on the rates of pulmonary embolism (PE). Six hundred and eighty-nine patients were treated with TXA and 702 with placebo (Fig. 5b). Of the 689 patients in the TXA group, seven were reported to have incidence of PE, whereas nine of the 702 patients in the placebo group had incidence of PE. A fixed model was applied. There was no difference between TXA and placebo groups in occurrence of PE (OR 0.82, 95% CI 0.33, 2.05, *I*^2^ = 0%).

#### Mortality

Eight studies involving 1494 patients reported on the events of death occurring postoperatively. Seven hundred and forty-three patients were treated with TXA and 751 with placebo (Fig. 5c). A fixed model was applied. There were 55 deaths reported in the TXA group compared to the 44 deaths in the control. There was no difference between TXA and placebo in terms of mortality (OR 1.27, 95% CI 0.84, 1.91, *I*^2^ = 0%).

## Discussion

This meta-analysis included eighteen studies consisting of 2350 patients, who underwent hip surgeries after hip fractures and were either administered TXA or placebo. Findings demonstrated that TXA reduced the amount of total and intraoperative blood loss, decreased rates of blood transfusion as well as levels of hemoglobin level decline postoperatively both in day 1 and 3, in comparison with the placebo. In addition, the risk of thromboembolic events occurring, such as DVT and PE, demonstrated no differences between the two groups. Length of hospital admission was also shown to be shorter in the TXA groups. Together, these results are consistent with the literature in continuing to demonstrate the feasibility of TXA as a safe and efficacious option in the surgical management of hip fracture patients.

Blood loss reduction is the primary parameter used to measure the efficacy of TXA in this meta-analysis. Formulas to calculate hidden and total blood loss have been described previously [21–24], with Nadler’s formula being the most frequently used. This meta-analysis found that intervention with TXA favored a reduced blood loss in comparison with placebo, which is consistent with findings from previous meta-analysis [13, 14]. However, there was heterogeneity in the calculation of total blood loss, ranging from 279.35 [25] to 902.4 mL [26] in the TXA group and from 417.89 [25] to 1226.3 mL [26] in the placebo group. Similarly, the hidden blood loss using Nadler’s [21] formula, which can be reported up to six times the amount measured from drainage instruments during surgery [27], also showed a trend of variability. Eleven of the eighteen studies reported hidden blood loss volume; therefore, more studies are needed to investigate whether the discrepancies in other measurements for blood loss can be accounted for by the hidden blood loss volume. While it is challenging to isolate these specific factors, there is no doubt characteristics of the patient, type of surgery and fracture as well as hospital protocols are all likely to contribute to this heterogeneity found in measuring blood loss. As such, future studies should place efforts to derive a gold standard method of providing more precise approximation of surgical blood loss [28]. Despite these discrepancies, the results from this meta-analysis clearly demonstrates that intervention with TXA reduces the amount of blood loss when compared to the placebo.

Another useful way of assessing the hemostatic efficacy of TXA is by examining the transfusion rate. Historically, the literature shows that TXA decreases the need for blood transfusions compared to control groups. This meta-analysis continues to exhibit these findings with TXA requiring a transfusion rate of 20.86% compared to 35.8% reported in a previous meta-analysis [13]. While there was no significant heterogeneity in the calculated difference in transfusion rates between the two groups, many of the included studies had their own transfusion criteria based on the hemoglobin level postoperatively. For example, Tian et al. [29] and Huang et al. [30] were given blood transfusion if the Hb level was < 9 mg/dl, while the transfusion trigger point set by Zhang et al., [31] was < 7 mg/dl. Thus, standardization of the transfusion criteria could help establish more reliable interpretation of the pooled data, but we also understand that patients that suffered from other comorbidities including anemia might need separate evaluation, as this was considered by Zhang et al. [31]. In addition to transfusion rates, the mean Hb level decline was reportedly lower in the intervention TXA groups at days 1 which was recorded as 102.13 g/L compared to the placebo, 97.05 g/L. Similarly, at day 3, the Hb level of the TXA group was 92.13 g/L, while the placebo reported 85.45 g/L. These findings are consistent with previous meta-analyses that investigated postoperative hemoglobin decline, all of which showed a decrease with TXA [14, 15]. Unlike the transfusion rates, the heterogeneity in Hb levels was high, which could have been due to factors such as the presence of comorbidities, the extent of blood loss incurred from surgery and the accuracy of Hb measurement postoperatively. Eight studies reported on Hb levels at day 1, while only six studies for day 3, which implies that a larger sample size is favorable for more accurate estimates of this outcome. Importantly, the findings from this meta-analysis continues to support the literature in demonstrating the ability of TXA to reduce both the rate of blood transfusions and the postoperative hemoglobin decline at days 1 and 3.

TXA has been shown to reduce postoperative complications such as renal failure and thromboembolic events [32, 33]. Importantly, thromboembolic events such as DVT and PE have been widely reported to be the most common complication of hip replacement surgeries. Obstruction of blood flow in the femoral vein caused by surgical maneuvers used to implant hip prosthetics along with the prolonged periods of immobilization after surgery can alter patients’ venous system increasing their risk of DVT/PE [33]. While there has been skepticism surrounding the use of TXA due to earlier reports of increasing the hypercoagulable state [16], our results are consistent with previous meta-analyses in showing no difference in the risk of thromboembolic events occurring between the two groups [13–15, 34]. Moreover, the clinical importance of reducing complications is reflected in the length of hospital stay. In the case of this meta-analysis, the TXA group was seen to have a reduced hospital stay length, thus making the susceptibility of postoperative complications such as infections less likely. Only a minority of infections are directly associated with wound infections from the surgical incision, whereas blood transfusions, in particular homologous blood transfusions has an infection rate of 32% compared to 3% in autologous blood transfusion [35]. This explains why there are less infections in TXA patients as the need for transfusions is reduced. Yazdi et al. [36] also reported that TXA reduces the occurrence of periprosthetic joint infection, which is a rare but very serious complication that can arise from total joint arthroplasties. Risks of wound complications were also reported to be less in the TXA groups when compared to the control from their study. Together, these results establish the safety of TXA to favor the likelihood of less postoperative infections and a similar risk of thromboembolic events from occurring.

The reported dosage of TXA can vary widely according to the protocols set out by various centers. The concentration recommended in orthopedic surgeries has no international consensus, but an optimum intravenous dose is believed to exist between 10 and 20 mg/kg or a fixed dose of 1–2 g [37]. In this meta-analysis, seven of the eighteen studies used a fixed dose of 1 g TXA, while other studies administered different weighted doses between10 mg/kg, 15 mg/kg, and 20 mg/kg. Levine et al., found no significant difference in TXA efficacy to reduce postoperative and total blood loss between a fixed 1 kg dose and 20 mg/kg weighted dose in knee arthroplasty [38]. Although the ability to reduce blood loss was found to be the same, more patients on the fixed dose required a transfusion compared to those given a weighted dose [38]. Further research is needed to clarify whether this would be similar for 10 mg/kg and 15 m/kg doses and how it would ultimately affect outcomes for patients with hip arthroplasty. Most studies in the meta-analysis gave a single dose of TXA five to fifteen minutes before commencing surgery. The literature shows there is no significant difference in either mean perioperative blood loss or transfusion rate between administering a single bolus and an infusion of TXA during surgery [39]. Therefore, a single dose of TXA is enough to both reduce blood loss and also to limit transfusion rates and the number of units required for each transfusion. Studies have also demonstrated that the risk of complications is lower with a single dose of TXA compared to a control, particularly asymptomatic distal DVT and other complications like myocardial infarction [40]. Administration of topical TXA has also been applied clinically and seen in the literature. Alshryda et al., showed decreased rates of blood transfusion and no differences in the rates of thromboembolic events occurring when patients were administered topical TXA, in comparison with the control [41]. Our meta-analysis excluded patients with topical administration as a larger sample size and more clinical trials are required to assess the efficacy of topical TXA. Furthermore, inclusion of studies that measured the effect of topical administration of TXA could have increased the degree of heterogeneity, thus future studies could explore these outcomes further with respect to hip fracture surgeries.

This meta-analysis was not devoid of limitations. Firstly, there was heterogeneity in primary and secondary outcomes, which could have been accounted for by the varying nature of surgical protocols and transfusion criteria set out across the different centers and institutes. Secondly, this meta-analysis did not isolate based on the type of hip fracture or type of surgery performed like previous meta-analysis which only focused on intertrochanteric fractures [42]. While this allows a wider examination on the efficacy of TXA, by including a larger sample size, it also introduced heterogeneity that could not be minimized due to the variability in amounts of blood loss and transfusion rates experienced by each individual based on the nature of their fracture and selected surgical management. The wide variation in these preexisting medical comorbidities, given the mean age of patients admitted for surgery (ranging from 67.6 to 84.3 years), may have also led to higher degrees of heterogeneity in primary outcomes. As described earlier, the amount of single bolus dose, loading doses and subsequent follow-up doses also varied between the studies, with the majority having a single dose. While there is no international consensus on the agreed amount of TXA to be administered for hip fracture surgeries, future studies could utilize a more consistent protocol to best assess and compare the effect of TXA on hip fracture surgeries. Furthermore, longer follow-up periods could have better examined any adverse effects of TXA. While these limitations may have impacted the quality of this meta-analysis, there is substantial evidence that tranexamic acid deserves an unbiased evaluation as a safe and efficacious agent in the management of hip fracture surgeries.

## Conclusion

From this study, administration of TXA intravenously shows an ability to reduce the amount of total blood loss and need for blood transfusions in patients undergoing hip fracture surgeries. Importantly, TXA did not show a greater risk of developing thromboembolic events from occurring when compared to the placebo. The results indicated heterogeneity due to the variability across the included studies; therefore, further studies are needed to validate the findings found in this meta-analysis. Nevertheless, the combination of results from this study are consistent with trends from the literature in demonstrating TXA as a safe and efficacious pharmacological agent in the management of patients undergoing hip fracture surgeries.

## Supplementary Information

Below is the link to the electronic supplementary material.

Supplementary file1 (DOCX 20 KB)

Supplementary file2 (DOCX 225 KB)

Supplementary file3 (DOC 65 KB)
