# Correction to: Update on the efficacy and safety of intravenous tranexamic acid in hip fracture surgery: a systematic review and meta‑analysis

**DOI:** 10.1007/s00590-023-03709-5

**Published:** 2023-09-07

**Authors:** Shahid Miangul, Timothy Oluwaremi, Joe El Haddad, Maamoun Adra, Nathan Pinnawala, Hayato Nakanishi, Reem H. Matar, Christian A. Than, Thomas M. Stewart

**Affiliations:** 1 St George’s University of London, London, SW17 0RE UK; 2 University of Nicosia Medical School, University of Nicosia, 2417 Nicosia, Cyprus; 3 Department of Gastroenterology and Hepatology, Mayo Clinic, Rochester, MN USA; 4 School of Biomedical Sciences, The University of Queensland, St Lucia, Brisbane, 4072 Australia; 5 Department of Anesthesiology and Perioperative Medicine, Mayo Clinic, Rochester, MN USA

**Correction to: European Journal of Orthopaedic Surgery & Traumatology (2023) 33:2179–2190** **https://doi.org/10.1007/s00590-022-03387-9**

The original version of this article unfortunately contained a mistake. In Table 3, incorrect numerical values were inserted into the table cells “Incidence of Post-Operative DVT (n)” and “Incidence of Post-Operative PE (n)”, which resulted in a discrepancy between the data presented in Table 3.

**Table 3:** Intraoperative and postoperative outcomes

^Outcomes^	^Placebo Group^	^TXA group^
^Total blood loss (mL)^	^807.30 ± 389.75^	^618.91 ± 282.72^
^Hidden blood loss (mL)^	^619.47 ± 311.93^	^438.86 ± 265.61^
^Intraoperative blood loss (mL)^	^136.09 ± 86.19^	^103.60 ± 75.75^
^Transfusion rate (n)^	^456 (n = 1118)^	^232 (n = 1112)^
^Post-operative Hb level day 1 (g/L)^	^95.55 ± 28.17^	^104.42 ± 13.72^
^Post-operative Hb level day 3 (g/L)^	^84.30 ± 17.79^	^91.46 ± 14.74^
^Incidence of post-operative DVT (n)^	^28 (n = 830)^	^34 (n = 831)^
^Incidence of post-operative PE (n)^	^9 (n = 702)^	^7 (n = 689)^
^Length of hospital stay (Days)^	^11.19 ± 5.80^	^10.13 ± 4.11^
^Operation time (Minutes)^	^80.30 ± 32.07^	^79.99 ± 30.96^

*DVT* deep vein thrombosis, *Hb* hemoglobin, *PE* pulmonary embolism

The corrected Table 3 should read as:

