# Supplementary material for: Update on the efficacy and safety of intravenous tranexamic acid in hip fracture surgery: a systematic review and meta-analysis
Source: Eur J Orthop Surg Traumatol. 2022 Sep 26;33(5):2179–90. doi: 10.1007/s00590-022-03387-9 (PMC10275812; doi:10.1007/s00590-022-03387-9)
Supplement: Supplementary file 1 — Supplementary file1 (DOCX 20 KB) [file 590_2022_3387_MOESM1_ESM.docx]

**Update on the efficacy and safety of intravenous tranexamic acid in hip fracture surgery: A systematic review and meta-analysis**

Shahid Miangul BSc^1,2^, Timothy Oluwaremi MSc^1,2^, Joe El Haddad BSc^1,2^, Maamoun Adra BSc^1,2^, Nathan Pinnawala BSc^1,2^, Hayato Nakanishi MSc^1,2^, Reem H. Matar BSc^1,2,3^, Christian A. Than PhD^1,2,4^ Thomas M. Stewart MD^5^

1 St George's University of London, London SW17 0RE, UK

2 University of Nicosia Medical School, University of Nicosia, 2417, Nicosia, Cyprus

3 Department of Gastroenterology and Hepatology, Mayo Clinic, Rochester MN

4 School of Biomedical Sciences, The University of Queensland, St Lucia, 4072, Brisbane, Australia

5 Department of Anesthesiology and Perioperative Medicine, Mayo Clinic, Rochester MN

**Corresponding author:**  Thomas M. Stewart MD

**Email address of corresponding author:**  Stewart.Thomas1@mayo.edu

**Supplementary Item 1**. Actual Search Engine

**Searches run 7/14/2021; updated 11/11/2021**

**OVID**

Database(s): **EBM Reviews - Cochrane Central Register of Controlled Trials** October 2021, **EBM Reviews - Cochrane Database of Systematic Reviews** 2005 to November 04, 2021, **Embase** 1974 to 2021 November 10, **Ovid MEDLINE(R) ALL** 1946 to November 10, 2021

| **#** | **Searches** | **Results** |
| --- | --- | --- |
| 1 | tranexamic acid/ or tranexamic acid.mp. or TXA.mp. or AMCHA.mp. or trans-4-Aminomethyl cyclohexanecarboxylic Acid.mp. or t-AMCHA.mp. or AMCA.mp. or Anvitoff.mp. or Cyklokapron.mp. or Ugurol.mp. or KABI 2161.mp. or Transamin.mp. or Exacyl.mp. or Spotof.mp. or Amchafibrin.mp. | 27433 |
| 2 | hip fracture/ or hip fractures/ or hip fracture.mp. or hip fractures.mp. | 66799 |
| 3 | femoral neck fracture/ or femoral neck fractures/ or femoral neck fracture.mp. or femoral neck fractures.mp. | 20363 |
| 4 | femur subtrochanteric fracture/ or subtrochanteric fracture.mp. or subtrochanteric fractures.mp. | 3303 |
| 5 | femur trochanteric fracture/ or trochanteric fracture.mp. or trochanteric fractures.mp. | 3786 |
| 6 | femur pertrochanteric fracture/ or pertrochanteric fracture.mp. or pertrochanteric fractures.mp. | 1809 |
| 7 | femur intertrochanteric fracture/ or intertrochanteric fracture.mp. or intertrochanteric fracture.mp. | 4441 |
| 8 | 2 or 3 or 4 or 5 or 6 or 7 | 87775 |
| 9 | 1 and 8 | 353 |
| 10 | remove duplicates from 9 | **253** |

**PubMed, 93 results**

("Tranexamic Acid"[Mesh] OR tranexamic acid [tiab] OR TXA [tiab] OR AMCHA OR “trans-4-(Aminomethyl)cyclohexanecarboxylic Acid” OR t-AMCHA OR AMCA OR Anvitoff OR Cyklokapron OR Ugurol OR KABI 2161 OR Transamin OR Exacyl OR Spotof OR Amchafibrin) AND ("Hip Fractures"[Mesh] OR hip fracture [tiab] OR hip fractures [tiab] OR hip fracture OR "Femoral Neck Fractures"[Mesh] OR femoral neck fracture [tiab] OR femoral neck fractures [tiab] OR subtrochanteric fracture [tiab] OR subtrochanteric fractures [tiab] OR trochanteric fracture [tiab] OR trochanteric fractures [tiab] OR pertrochanteric fracture [tiab] OR pertrochanteric fractures [tiab] OR intertrochanteric fracture [tiab] OR intertrochanteric fractures [tiab])

**Web of Science, 92 results**

(“tranexamic acid” OR TXA OR AMCHA OR “trans-4-(Aminomethyl)cyclohexanecarboxylic Acid” OR “t-AMCHA” OR AMCA OR Anvitoff OR Cyklokapron OR Ugurol OR KABI 2161 OR Transamin OR Exacyl OR Spotof OR Amchafibrin) AND (“hip fracture” OR “hip fractures” OR “femoral neck fracture” OR “femoral neck fractures” OR “subtrochanteric fracture” OR “subtrochanteric fractures” OR “trochanteric fracture” OR “trochanteric fractures” OR “pertrochanteric fracture” OR “pertrochanteric fractures” OR “intertrochanteric fracture” OR “intertrochanteric fractures”)

438 total article references, 167 duplicates found in EndNote, **271 total references in EndNote**

**Clinicaltrials.gov**

<https://clinicaltrials.gov/ct2/results?cond=tranexamic+acid+AND+hip+fracture&term=&cntry=&state=&city=&dist=>

search: tranexamic acid AND hip fracture

**Google Scholar**

<https://scholar.google.com/scholar?hl=en&as_sdt=0%2C24&inst=12058184521150304743&q=%28%E2%80%9Ctranexamic+acid%E2%80%9D+OR+TXA+OR+AMCHA+OR+%E2%80%9Ctrans-4-%28Aminomethyl%29cyclohexanecarboxylic+Acid%E2%80%9D+OR+%E2%80%9Ct-AMCHA%E2%80%9D+OR+AMCA+OR+Anvitoff+OR+Cyklokapron+OR+Ugurol+OR+KABI+2161+OR+Transamin+OR+Exacyl+OR+Spotof+OR+Amchafibrin%29+AND+%28%E2%80%9Chip+fracture%E2%80%9D+OR+%E2%80%9Chip+fractures%E2%80%9D+OR+%E2%80%9Cfemoral+neck+fracture%E2%80%9D+OR+%E2%80%9Cfemoral+neck+fractures%E2%80%9D+OR+%E2%80%9Csubtrochanteric+fracture%E2%80%9D+OR+%E2%80%9Csubtrochanteric+fractures%E2%80%9D+OR+%E2%80%9Ctrochanteric+fracture%E2%80%9D+OR+%E2%80%9Ctrochanteric+fractures%E2%80%9D+OR+%E2%80%9Cpertrochanteric+fracture%E2%80%9D+OR+%E2%80%9Cpertrochanteric+fractures%E2%80%9D+OR+%E2%80%9Cintertrochanteric+fracture%E2%80%9D+OR+%E2%80%9Cintertrochanteric+fractures%E2%80%9D%29&btnG=>

search: (“tranexamic acid” OR TXA OR AMCHA OR “trans-4-(Aminomethyl)cyclohexanecarboxylic Acid” OR “t-AMCHA” OR AMCA OR Anvitoff OR Cyklokapron OR Ugurol OR KABI 2161 OR Transamin OR Exacyl OR Spotof OR Amchafibrin) AND (“hip fracture” OR “hip fractures” OR “femoral neck fracture” OR “femoral neck fractures” OR “subtrochanteric fracture” OR “subtrochanteric fractures” OR “trochanteric fracture” OR “trochanteric fractures” OR “pertrochanteric fracture” OR “pertrochanteric fractures” OR “intertrochanteric fracture” OR “intertrochanteric fractures”)
