# Supplementary material for: Update on the efficacy and safety of intravenous tranexamic acid in hip fracture surgery: a systematic review and meta-analysis
Source: Eur J Orthop Surg Traumatol. 2022 Sep 26;33(5):2179–90. doi: 10.1007/s00590-022-03387-9 (PMC10275812; doi:10.1007/s00590-022-03387-9)

**Update on the efficacy and safety of intravenous tranexamic acid in hip fracture surgery: A systematic review and meta-analysis**

Shahid Miangul BSc^1,2^, Timothy Oluwaremi MSc^1,2^, Joe El Haddad BSc^1,2^, Maamoun Adra BSc^1,2^, Nathan Pinnawala BSc^1,2^, Hayato Nakanishi MSc^1,2^, Reem H. Matar BSc^1,2,3^, Christian A. Than PhD^1,2,4^ Thomas M. Stewart MD^5^

1 St George's University of London, London SW17 0RE, UK

2 University of Nicosia Medical School, University of Nicosia, 2417, Nicosia, Cyprus

3 Department of Gastroenterology and Hepatology, Mayo Clinic, Rochester MN

4 School of Biomedical Sciences, The University of Queensland, St Lucia, 4072, Brisbane, Australia

5 Department of Anesthesiology and Perioperative Medicine, Mayo Clinic, Rochester MN

**Corresponding author:**  Thomas M. Stewart MD

**Email address of corresponding author:**  Stewart.Thomas1@mayo.edu


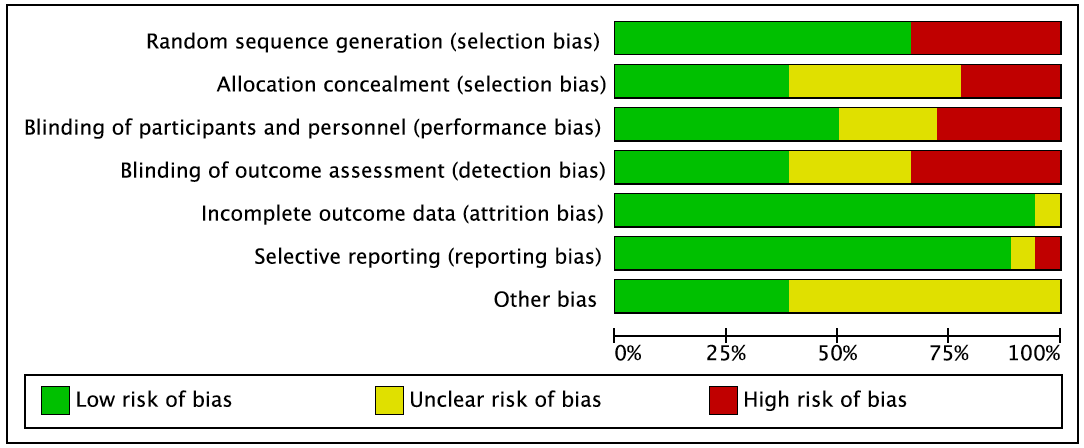


**Supplementary Item 2.** Cochrane Tool for Assessing Risk Bias


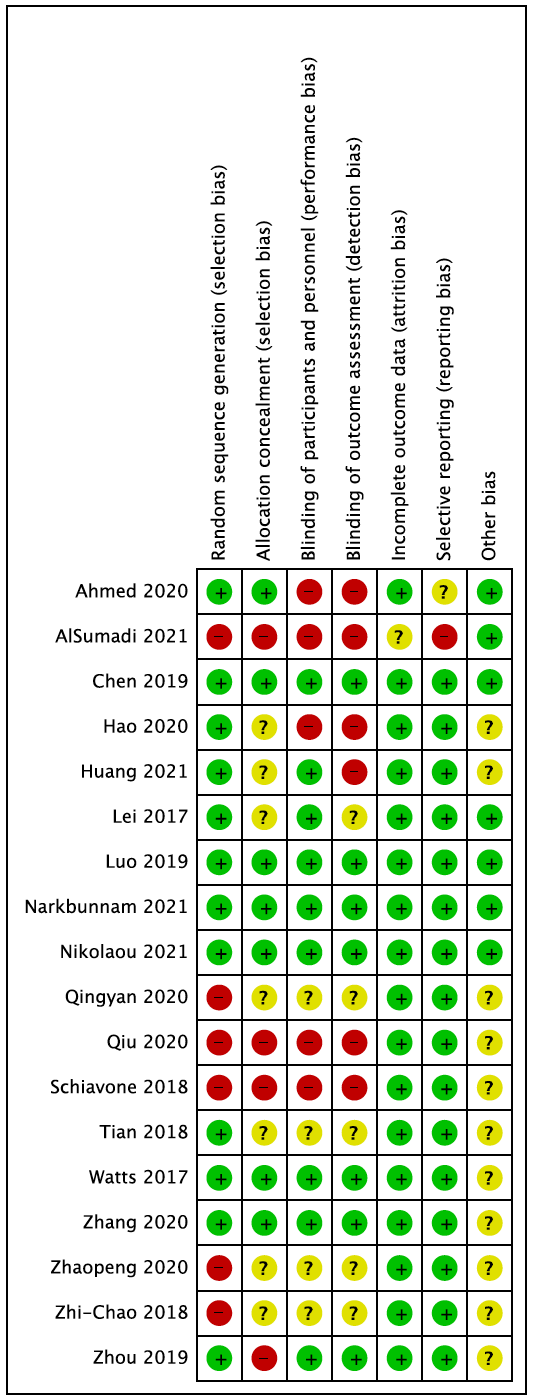

Supplement: Supplementary file 2 — Supplementary file2 (DOCX 225 KB) [file 590_2022_3387_MOESM2_ESM.docx]
