# Supplementary material for: Update on the efficacy and safety of intravenous tranexamic acid in hip fracture surgery: a systematic review and meta-analysis
Source: Eur J Orthop Surg Traumatol. 2022 Sep 26;33(5):2179–90. doi: 10.1007/s00590-022-03387-9 (PMC10275812; doi:10.1007/s00590-022-03387-9)
Supplement: Supplementary file 3 — Supplementary file3 (DOC 65 KB) [file 590_2022_3387_MOESM3_ESM.doc]

**Update on the efficacy and safety of intravenous tranexamic acid in hip fracture surgery: A systematic review and meta-analysis**

Shahid Miangul BSc^1,2^, Timothy Oluwaremi MSc^1,2^, Joe El Haddad BSc^1,2^, Maamoun Adra BSc^1,2^, Nathan Pinnawala BSc^1,2^, Hayato Nakanishi MSc^1,2^, Reem H. Matar BSc^1,2,3^, Christian A. Than PhD^1,2,4^ Thomas M. Stewart MD^5^

1 St George's University of London, London SW17 0RE, UK

2 University of Nicosia Medical School, University of Nicosia, 2417, Nicosia, Cyprus

3 Department of Gastroenterology and Hepatology, Mayo Clinic, Rochester MN

4 School of Biomedical Sciences, The University of Queensland, St Lucia, 4072, Brisbane, Australia

5 Department of Anesthesiology and Perioperative Medicine, Mayo Clinic, Rochester MN

**Corresponding author:**  Thomas M. Stewart MD

**Email address of corresponding author:**  Stewart.Thomas1@mayo.edu

**Supplementary Item 3.** PRISMA Flowchart


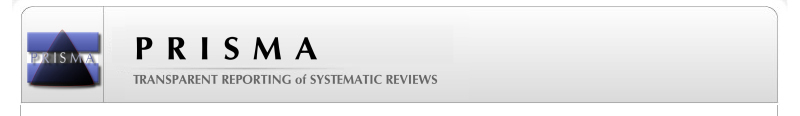
**PRISMA 2009 Flow Diagram**

**Screening**

**Included**

**Eligibility**

**Identification**

Records identified through database searching
(n = 438)

Additional records identified through other sources
(n = 0)

Records after duplicates removed
(n = 271)

Records screened
(n = 271)

Records excluded
(n = 217)

Full-text articles assessed for eligibility
(n = 54)

Full-text articles excluded, according to exclusion criteria
(n = 36)

b

Studies included in qualitative synthesis
(n = 18)

Studies included in quantitative synthesis (meta-analysis)
(n = 18)
